# Effects Resulting from the Domestic Use of Champignons in France

**Published:** 1809-12

**Authors:** 


### *Effects resulting from the Domestic Use of Champignons in France.*

IN the communes of Portels, an old woman, in the habit of gathering champignons for sale, proceeded to the fir woods in that neighbourhood on the 11th of October last, with a view to collect her winter's store. She gathered a kind with which she was quite familiar, and ate of them all three successive meals, namely, supper, breakfast, and dinner. On this last occasion, a young woman, her neighbour, who visited her by accident, requested and obtained a few of the champignons, in order to regale her husband and family.

The husband, wife, and a daughter ate of these champignons at dinner, and continued their usual occupations without feeling any pain: at midnight, however, nausea, pains in the bowels, debility, and other symptoms of poison were manifested in all three individuals. But having no suspicion that the champignons produced these disagreeable effects, they did not call in a surgeon until forty-eight hours afterwards, that is to say, when the poison had effected its purpose, and the inflammation of the primæ viæ rendered all attempt at relief fruitless; in short, these three persons died within a few hours of each other.—The following is the most extraordinary part of the case.

The old woman, who had first eaten so heartily of the champignons, refused to take a vomit, or any of the antidotes prescribed by the surgeon who was called in; and yet she completely resisted every poisonous effect, with the exception of some acute pains in the kidnies and intestines, and and a great debility. This patient was 73 years of age; had been long infirm, and her stomach was entirely worn out and tattered, (*usé et delabré*) while the three individuals who died, were healthy, vigorous, and in the flower of their age.

The following solution of this singular phænomenon, has been offered by the surgeon who attended all the cases.

The old woman, whom the poison seemed to have respected, was addicted to drinking: she was in a state of intoxication on the three occasions when she swallowed the champignons, and this state of drunkenness saved her life. The wine which she drank was sour, and this remarkable circumstance leaves no uncertainty as to the efficacy of acids against similar poisons.

The champignons which caused the death of the three persons above-mentioned, were of two kinds: the first, known by the name of *Pinadossa*, grow at the roots of fir trees; they are fungous, similar to what are produced on the old roots of the alder and poplar trees; the second, known in that part of France by the name of *Catalans*, is of a deep red colour; it is membranous below; the stalk is short and hollow, (*cannelè.*) Those who died in consequence of eating them, as well as every person who saw and ecxamined them, stated that they had eaten them repeatedly in former years, without the least bad consequences.

It would seem to follow, that champignons benign in their nature, may become poisonous in seasons like the present, when the genial influence of the sun has been interrupted by continual fogs, rains, or damps.

*Moniteur, Nov.* 9 1809.

